# Measles outbreak in Gothenburg urban area, Sweden, 2017 to 2018: low viral load in breakthrough infections

**DOI:** 10.2807/1560-7917.ES.2019.24.17.1900114

**Published:** 2019-04-25

**Authors:** Nicklas Sundell, Leif Dotevall, Martina Sansone, Maria Andersson, Magnus Lindh, Thomas Wahlberg, Tobias Tyrberg, Johan Westin, Jan-Åke Liljeqvist, Tomas Bergström, Marie Studahl, Lars-Magnus Andersson

**Affiliations:** 1 Department of Infectious Diseases, Institute of Biomedicine, Sahlgrenska Academy, University of Gothenburg, Gothenburg, Sweden; 2 Department of Communicable Disease Control, Region Västra Götaland, Gothenburg, Sweden

**Keywords:** measles, outbreak, PCR, vaccine, immunity, serology

## Abstract

In an outbreak of measles in Gothenburg, Sweden, breakthrough infections (i.e. infections in individuals with a history of vaccination) were common. The objective of this study was to compare measles RNA levels between naïve (i.e. primary) and breakthrough infections. We also propose a fast provisional classification of breakthrough infections. Medical records were reviewed and real-time PCR-positive samples genotyped. Cases were classified as naïve, breakthrough or vaccine infections. We compared clinical symptoms and measles RNA cycle threshold (Ct) values between breakthrough and naïve infections. Sixteen of 28 confirmed cases of measles in this outbreak were breakthrough infections. A fast provisional classification, based on previous history of measles vaccination and detectable levels of measles IgG in acute serum, correctly identified 14 of the 16 breakthrough infections, confirmed by IgG avidity testing. Measles viral load was significantly lower in nasopharyngeal samples from individuals with breakthrough compared with naïve infections (median Ct-values: 32 and 19, respectively, p < 0.0001). No onward transmission from breakthrough infections was identified. Our results indicate that a high risk of onward transmission is limited to naïve infections. We propose a fast provisional classification of breakthrough measles that can guide contact tracing in outbreak settings.

## Introduction

The 2-dose regime of measles vaccination has greatly reduced the morbidity and mortality of measles [1,2]. Measles vaccination has been offered to all children in Sweden since 1971, and a 2-dose regimen of measles-mumps-rubella (MMR) vaccination was introduced in the Swedish national child vaccination programme in 1982. The first MMR vaccination is currently scheduled at 18 months and the second at 6–8 years of age. The average vaccine coverage has been estimated to exceed 95% in children 2 years or older and in a seroprevalence study from 2007, protective levels of antibodies against measles were found in 98% of participants [3,4].

There have been reports of measles in previously immunised individuals, especially in healthcare workers (HCW) [5-10]. In areas with high vaccination coverage, it has been estimated that the majority of cases in an outbreak will be breakthrough infections (i.e. infections in individuals with a history of vaccination) [11,12]. Transmission of infection from individuals with breakthrough infections seems to be rare [10,13-15], and the recently published national measles guidelines by Public Health England recommend only limited contact tracing around such cases [16]. The current Swedish guidelines recommend extensive contact tracing around all confirmed cases of measles, leading to substantial contact tracing efforts [4]. Breakthrough infections cannot be distinguished from naïve (i.e. primary) infections based on clinical presentation alone [13]. Measles-specific IgG antibody titres are usually high in acute-phase serum but IgM may be undetectable, making analysis of measles virus RNA by real-time PCR the preferred diagnostic method for breakthrough infections [17,18]. Analysis of measles IgG antibody avidity or measles neutralising antibodies by plaque reduction neutralisation assays (PRN) in acute serum samples are used to confirm breakthrough infections [10,15,19,20].

The aim of this study was to report an outbreak of measles, with focus on the real-time PCR results in nasopharyngeal, urine and serum samples in individuals without pre-existing immunity in comparison with subjects with breakthrough infections. Based on the observations, we propose a fast provisional classification of breakthrough infections that may guide decisions regarding contact tracing and infection control during an outbreak.

### Outbreak description

**Figure 1:**
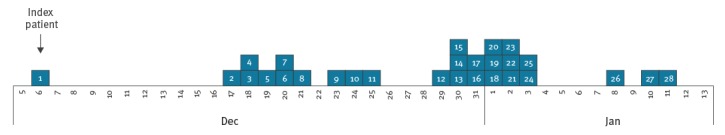
Confirmed cases of measles during the outbreak in the Gothenburg area, by date of symptom onset, Sweden, December 2017–January 2018 (n = 28)

**Figure 2:**
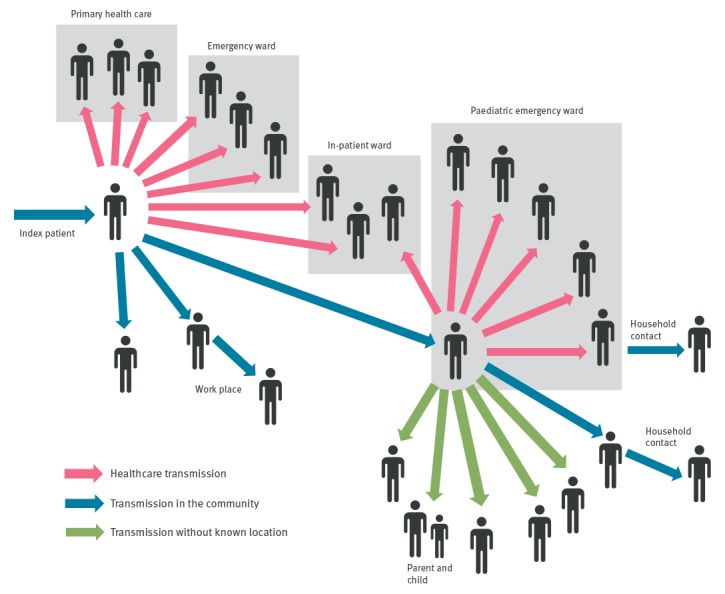
Transmission chains during the measles outbreak in the Gothenburg area, Sweden, December 2017– January 2018 (n = 28)

On 10 December 2017, a young adult was admitted to Sahlgrenska University hospital in Gothenburg, Sweden, with a 4-day history of fever and a generalised rash for 2 days. Measles infection was confirmed by real-time PCR. More than 300 individuals were regarded as potentially exposed and a large-scale contact tracing effort among exposed individuals in the community, patients and HCW was initiated. Between December 2017 and February 2018, an additional 27 cases of measles were diagnosed in two major transmission chains (Figure 1 and 2) in the Gothenburg area (with a population of approximately one million inhabitants).

## Methods

We performed a retrospective review of medical records and laboratory results of laboratory-confirmed cases of measles.

### Definitions

The European Union’s (EU) case definitions for measles and laboratory criteria are used in Sweden [21]. During the outbreak, the definition of a suspected case was a possibly non-immune person with possible exposure to measles and at least one of the following symptoms: fever, maculopapular rash, conjunctivitis or respiratory symptoms [4]. For contact tracing, a contact was defined according to Swedish guidelines as an individual who had spent time indoors together with a laboratory-confirmed case of measles in the period 4 days before to 4 days after the onset of rash [4].

Naïve (i.e. primary) infection was defined as a confirmed case of measles in an immunologically naïve individual with no evidence of pre-existing immunity (no history of immunisation or measles infection and negative titres of measles-specific IgG antibodies in acute serum at or after onset of rash (if taken within 4 days), regardless of whether post-exposure prophylaxis (vaccine or immunoglobulin) was given or not.

Breakthrough infection was defined as a confirmed case of measles in an individual with history of vaccination and/or positive IgG levels (> 399 mIU/mL) in acute serum at or after onset of rash (if taken within 4 days), regardless of whether post-exposure prophylaxis was given or not. A fast provisional classification was established that included an individual with history of vaccination (not necessarily documented) and positive IgG levels (> 399 mIU/mL) in acute serum at or after onset of rash (if taken within 4 days). A confirmed breakthrough infection was defined as a case of confirmed measles with high-avidity IgG antibodies in acute serum according to criteria from the World Health Organization (WHO) Global Measles and Rubella Laboratory Network [22].

Vaccine infection was defined as an individual with rash but without respiratory symptoms and a history of measles vaccination 7–14 days before rash onset according to the definition by the WHO Global Measles and Rubella Laboratory Network [22]. As a large proportion of individuals received pre- and post-exposure prophylaxis during the outbreak, detection of measles RNA in nasopharyngeal, urine or blood samples and confirmation of infection with the vaccine strain by genotyping was included in the definition.

### Patient characteristics

Demographic characteristics as well as clinical data and medical history were obtained from medical records. Previous measles infection, history of measles immunisation, the number of vaccine doses (based on review of vaccination booklets when available or self-reporting) and post-exposure prophylaxis were registered.

All confirmed cases (older than 1 year) were offered a follow-up visit (FU) 4-8 weeks after infection for serum sampling and analysis of IgM and IgG levels. Cases were categorised into three subgroups: naïve infection, breakthrough infection and vaccine infection.

### Measles antibody assays

Acute and convalescent sera were tested in parallel using the Enzygnost (Siemens Healthcare Diagnostics Products, Eschborn, Germany) anti-measles IgG and IgM enzyme immunoassays on a BEP 2000 ELISA robot (Siemens Healthcare Diagnostics Products). Samples with IgG antibody levels > 399 mIU/mL were considered positive.

Avidity of IgG antibodies against measles virus was tested by a commercial test (Euroimmun, Avidity determination of antibodies against measles virus (IgG), Order no El 2610–9601–1G, Medizinische Labordiagnostika AG, Lübeck, Germany). In measles IgG-positive samples, results were calculated as relative avidity index (RAI). RAI < 40% was defined as low avidity, 40–60% as equivocal and > 60% as high avidity.

### Detection of measles virus RNA and sequencing

Real-time PCR was used to detect measles virus RNA in nasopharyngeal, urine and serum samples [23]. Nucleic acid from 200 µL specimens was extracted by a MagNA Pure LC instrument (Roche Diagnostics, Mannheim, Germany) using the Total Nucleic Acid isolation kit. The nucleic acids were eluted in 100 μL volume and 10 μL of this were used for real-time PCR, which was performed in an ABI 7500 instrument (Applied Biosystems, Foster City, United States (US)) in 50 μL reaction volume containing primers (measN1F, CGATGACCCTGACGTTAGCA; measN1R, GCGAAGGTAAGGCCAGATTG) and probe (measN1P, AGGCTGTTAGAGGTTGTCCAGAGTGACCAG), and SuperScript III Platinum One-Step qRT PCR kit with ROX (Invitrogen). After a reverse transcription step at 46 °C for 30 min followed by 10 min of denaturation at 95 °C, 45 cycles of two-step real-time PCR was performed (15 s at 95 °C, 60 s at 58 °C). The cycle threshold (Ct) value was used as a semiquantitative measure of the viral load of measles virus RNA in the sample.

Measles virus genotyping was performed by sequencing of the C-terminal part of the nucleocapsid gene (N-450) at the national reference laboratory at the Public Health Agency of Sweden in Stockholm and the sequences were deposited by the reference laboratory in the WHO MeaNS database.

For additional distinction, a 400 nt segment of the hypervariable region (HVR) was amplified using primers MorbHVR_F1 (TTCCGCATTTACGACGACGTGA) and MorbHVR_R1 (GTTCCTTGGCCCTAAGTTTTGT). When needed, a second (inner) PCR was performed using primers MorbHVR_F2 (GTGATCATAAATGATGACCAAGGAC) and MorbHVR_R2 (GTCACCTCGGTCGCTTGTG). A cycle sequencing reaction was then performed using the same primers as used in the amplification.

The sequences were aligned with reference sequences from GenBank and phylogenetic analysis was performed using MEGA7 software [24]. A phylogenetic tree was created by maximum likelihood method based on the Tamura–Nei model after bootstrapping to 500 replicates.

### Statistical analysis

Continuous variables were compared using Mann–Whitney U-test and comparisons of proportions were made using Pearson’s chi-squared or Fischer’s exact tests as appropriate. P values < 0.05 were considered statistically significant. All statistical analyses were done with the SPSS software package version 22.0.0.0 (IBM, Armonk, New York, US).

### Ethical approval

The study was approved by the Research Ethics Committee at Gothenburg University (Dnr 409–18).

## Results

### Characteristics and classification of cases

Clinical characteristics and laboratory results for all 28 cases of measles including laboratory results from FU are presented in Tables 1 and 2. The median age was 32 years (interquartile range (IQR): 22–40) and 20 of the 28 cases were female. Nine of the cases were HCW. In 20 of the 28 cases, a FU serum sample was obtained 4–8 weeks after first sampling.

**Table 1:** Patient characteristics and laboratory results for patients with laboratory-confirmed measles during the outbreak in the Gothenburg area, Sweden, December 2017–January 2018 (n = 28)

Patient	Infection type^a^	Age group (years)	Doses of measles vaccine received	Days from onset of rash to sampling	Symptoms	Fulfilled EU criteria for confirmed case^b^	Infected others
1	N	21–30	0	3	R F K C Z	Yes	Yes
2	N	31–40	0	3	R F C	Yes	Yes
3	N	0–10	0	2	R F C Z	Yes	Yes
4	N	0–10	0	1	R F C	Yes	No
5	N	31–40	0	0	R F C	Yes	No
6	N	0–10	0	0	R F C	Yes	No
7	N	0–10	0	0	R F C	Yes	Yes
8	N	51–60	0	4	R F K C	Yes	Yes
9	N	31–40	0	4	R F C	Yes	No
10	N	0–10	0	1	R F K C	Yes	No
11	N	0–10	0	1	R F K C	Yes	No
12^c^	N	31–40	0	5	R F K C	Yes	No
13	B	21–30	1^d^	1	R F	No	No
14	B	31–40	2	1	R F	No	No
15	B	31–40	2	0	R F	No	No
16	B	31–40	2	0	R F	No	No
17	B	11–20	1^d^	0	R F	No	No
18	B	41–50	1^d^	1	R F C	Yes	No
19	B	31–40	2	1	R F K	Yes	No
20	B	51–60	1^d^	1	R F K C	Yes	No
21	B	31–40	1^d^	0	R F	No	No
22	B	21–30	1	1	R F	No	No
23	B	31–40	1	0	R F K Z	Yes	No
24	B	31–40	1	0	R F C	Yes	No
25	B	51–60	2	0	R	No	No
26	B	31–40	1^d^	2	R F	No	No
27	B	21–30	1^d^	3	R F	No	No
28	B	51–60	1	1	R F K C	Yes	No

B: breakthrough infection; C: cough; EU: European Union; F: fever; K: conjunctivitis; N: naïve infection; ND: not done; R: rash; Z: coryza.

^a^ Breakthrough infection was defined as a confirmed case of measles in an individual with history of vaccination and/or positive IgG levels (> 399 mIU/mL) in acute serum at or after onset of rash (if taken within 4 days), regardless of whether post-exposure prophylaxis was given or not.

^b^ Fulfilled both clinical and laboratory criteria according to the EU case definition.

^c^ Patient 12 had no history of vaccination against measles and no history of measles infection. This patient presented low levels of IgG (579 mIU/mL) at first sampling 5 days after onset of rash and had received post-exposure measles vaccine 7 days before onset of rash.

^d^ Reported at least one dose of measles vaccine, not documented.

**Table 2:** Patient characteristics and laboratory results for patients with laboratory-confirmed measles during the outbreak in the Gothenburg area, Sweden, December 2017–January 2018 (n = 28)

Patient	Infection type^a^	IgM acute sera	IgG (mIU/mL) acute sera	IgM FU	IgG (mIU/mL) FU	Ct value NP	Ct value urine	Ct value blood	Avidity index^b^ (%) acute sera	Avidity index^b^ (%) FU
1	N	Equivocal	Neg	Pos	6,191	18	21	Neg	NA	54 (E)
2	N	Pos	Neg	Pos	10,083	22	22	38	NA^c^	67 (HA)
3	N	Pos	Neg	ND	ND	17	20	17	NA	ND
4	N	Neg	Neg	ND	ND	28	ND	36	NA	ND
5	N	Pos	Neg	ND	ND	22	26	31	NA	ND
6	N	Equivocal	Neg	ND	ND	20	24	31	NA	ND
7	N	Pos	Neg	Equivocal	13,454	18	ND	ND	NA^c^	73 (HA)
8	N	Pos	Neg	ND	ND	19	17	32	NA	ND
9	N	Neg	Neg	Neg	3,648	22	ND	ND	NA	44 (E)
10	N	Pos	Neg	Equivocal	9,420	17	ND	ND	NA	58 (E)
11	N	Pos	Neg	Pos	12,682	18	22	32	NA	ND
12^d^	N	Neg	579	Neg	5,204	19	19	32	16 (LA)	45 (E)
13	B	Neg	11,952	Neg	29,730	33	33	Neg	> 99 (HA)	> 99 (HA)
14	B	Equivocal	22,650	Equivocal	> 30,000	35	38	Neg	94 (HA)	91 (HA)
15	B	Neg	7,040	Neg	> 30,000	25	35	Neg	80 (HA)	89 (HA)
16	B	Neg	508	Equivocal	26,891	32	31	Neg	65 (HA)	96 (HA)
17	B	Neg	2,513	Neg	> 30,000	34	34	ND	78 (HA)	92 (HA)
18	B	Equivocal	5,593	ND	ND	24	27	Neg	ND^e^	ND
19	B	Neg	27,960	Neg	> 30,000	31	29	Neg	99 (HA)	> 99 (HA)
20	B	Equivocal	29,425	Neg	28,030	Neg	35	Neg	96 (HA)	97 (HA)
21	B	Neg	3,931	Neg	> 30,000	Neg	34	Neg	85 (HA)	> 99 (HA)
22	B	Neg	4,350	Equivocal	> 30,000	37	29	Neg	82 (HA)	> 99 (HA)
23	B	Neg	Neg	ND	ND	31^f^	ND	ND	ND	ND
24	B	Equivocal	28,980	Neg	> 30,000	Neg	34	Neg	90 (HA)	97 (HA)
25	B	Neg	2,115	Equivocal	27,883	Neg	Neg	36	71 HA)	94 (HA)
26	B	Equivocal	209,570	Neg	> 30,000	Neg	34	Neg	94 (HA)	98 (HA)
27	B	Equivocal	192,190	Neg	> 30,000	31	Neg	Neg	91 (HA)	99 (HA)
28	B	Neg	Neg	ND	ND	37	39	40	ND^e^	ND

B: breakthrough infection; Ct: cycle threshold; E: equivocal; FU: follow-up visit; HA: high avidity; LA: low avidity; N: naïve infection; NA: not applicable; ND: not done; Neg: negative; NP: nasopharynx; Pos: positive.

^a^ Breakthrough infection was defined as a confirmed case of measles in an individual with history of vaccination and/or positive IgG levels (> 399 mIU/mL) in acute serum at or after onset of rash (if taken within 4 days), regardless of whether post-exposure prophylaxis was given or not.

^b^ Relative avidity index was calculated according to instructions by the manufacturer: HA > 60%, E 40–60%, LA < 40%.

^c^ Low levels of low-avidity IgG antibodies (below the detection limit of the standard IgG assay) were detected, in Patient 2 (31%) and in Patient 7 (23%).

^d^ Patient 12 had no history of vaccination against measles and no history of measles infection. This patient presented low levels of IgG (579 mIU/mL) at first sampling 5 days after onset of rash and had received post-exposure measles vaccine 7 days before onset of rash.

^e^ Not done because of insufficient material.

^f^ Performed at a different laboratory with the same method.

Twelve of the 28 cases were classified as naïve infections. Eleven of those had no history of vaccination and were negative for measles IgG at first sampling. Patient 12 had low levels of IgG (579 mIU/mL) 5 days after onset of rash, but had no history of vaccination against measles or of measles infection. This patient had received post-exposure measles vaccine 7 days before the onset of rash and was therefore considered to have a naïve infection, which was confirmed by genotyping and IgG avidity testing.

Sixteen of the 28 cases were identified as breakthrough infections. They had previously received at least one dose of measles vaccine (five had two and four had at least one dose of documented vaccination and 7 reported at least one dose of vaccination but this could not be confirmed by documentation), and all but two (Patient 23 and 28) had measles IgG > 399 mIU/mL at onset of rash. Our proposed fast provisional classification identified 14 of 16 breakthrough infections. They were confirmed by IgG avidity testing of acute serum samples (Table 1 and 2). Unfortunately, patient 23 and 28 did not agree to take a FU sample.

### Vaccine infections

In addition, six confirmed vaccine infections were diagnosed in adult patients, who all received their first dose of measles vaccine during or directly after the outbreak (Table 3). None of them had a history of measles vaccination before the outbreak. Vaccination was given as post- exposure prophylaxis, but also to individuals in the society who were uncertain of their vaccination history. The median age of the six persons was 42 years (IQR: 20–54). Four of six were female and one was HCW. The mean time from MMR vaccination to rash was 12.2 days (IQR: 11–13 days; n = 6) and the mean duration from vaccination to fever was 9.3 days (IQR: 8–10 days; n = 6).

**Table 3:** Patient characteristics and laboratory results in the six patients with vaccine infection in the Gothenburg area, Sweden, December 2017–January 2018 (n = 6)

Patient	Age group (years)	Days from onset of rash to sampling	Symptoms	IgM	IgG (mIU/mL)	Ct value NP	Ct value urine	Ct value blood	IgM-FU	IgG-FU (mIU/mL)
29	11–20	0	R F K C Z	Neg	Neg	32	33	38	Equivocal	2,750
30	31–40	0	R F K	Neg	Equivocal	32	30	40	Pos	4,027
31	41–50	1	R F C	Pos	Neg	19	31	38	ND	ND
32	51–60	0	R F	Neg	Neg	32	29	Neg	Neg	1,345
33	41–50	1	R F K C Z	Neg	Neg	Neg	ND	Neg	Neg	1,582
34	51–60	1	R F	Equivocal	Neg	26	Neg	39	ND	ND

C: cough; Ct: cycle threshold; F: fever; FU: follow-up visit; K: conjunctivitis; ND: not done; Neg: negative; NP: nasopharyngeal secrete; Pos: positive; R: rash; Z: coryza.

### Genotyping and sequencing

**Figure 3:**
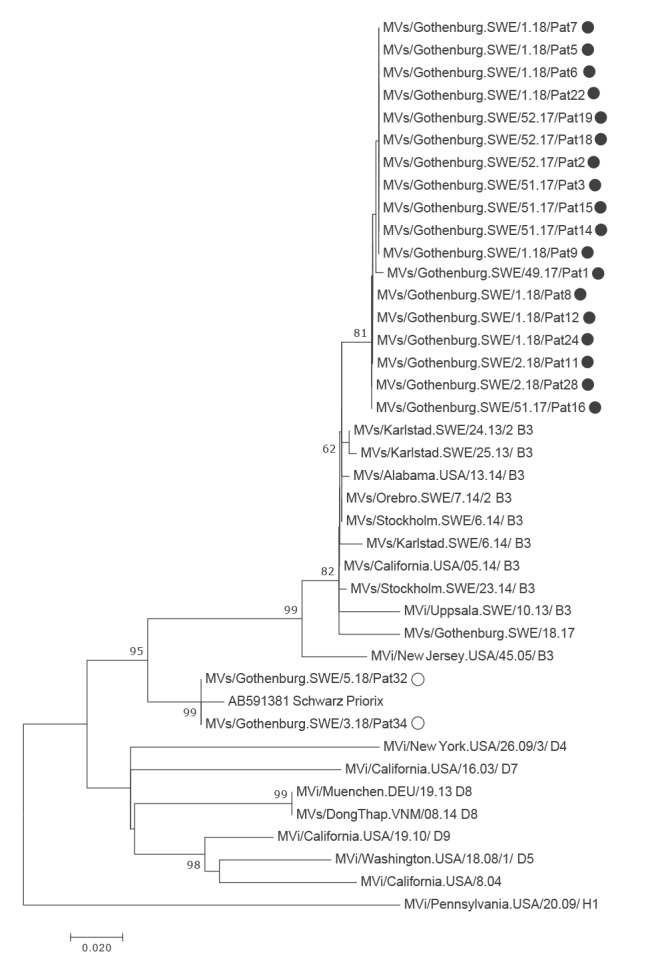
Phylogenetic tree based on maximum likelihood analysis of a 400 nt segment from the hypervariable region of measles virus, measles outbreak, Gothenburg, December 2017–January 2018 (n =20)

In 25 of 28 cases, it was possible to genotype the measles virus strain. All these cases were infected with subtype B3. Twenty strains were sequenced, also in the hypervariable region (HVR). Eighteen of them clustered together on the B3 branch with no or minimal genetic distance between the sequences. Two sequences, obtained from persons who had been vaccinated 11–13 days before sampling, clustered with the Priorix vaccine strain (Figure 3).

The tree includes 20 sequences from samples taken during the measles outbreak in Gothenburg, Sweden and a selection of sequences representing different subgenotypes and sampling time points, including viruses identified in Sweden during earlier outbreaks. Bootstrapping was done to 500 replicates, using the MEGA7 software. Black filled circles: 18 B3 strains; open circles: two vaccine strains. A distance scale bar is included.

### Comparison of clinical parameters between subgroups

The clinical presentation in patients with naïve and breakthrough infection are compared in Table 4. All cases presented with a maculopapular rash. Cases with naïve infections had a generalised rash and a clinical course suggestive of moderate to severe disease, whereas breakthrough infections had a mild or moderate rash and symptoms suggestive of mild to moderate disease. Six of the 16 breakthrough infections fulfilled the EU clinical case definition of a possible case. The number of patients with cough was lower in breakthrough infections (4/16) compared with naïve infections (12/12), (p < 0.0001).

**Table 4:** Clinical characteristics in patients with naïve and breakthrough measles infection in the Gothenburg area, Sweden, December 2017–January 2018 (n = 28)

Clinical features	Naïve infection (n = 12)			Breakthrough infection (n = 16)			p value^a^
	n	%	95% CI	n	%	95% CI	
Fever	12	100	70–100	15	94	68–100	1
Cough	12	100	70–100	4	25	8–53	< 0.0001
Conjunctivitis	5	42	17–71	4	25	8–53	0.4
Coryza	2	17	3–49	1	6	0.3–32	0.6
Sore throat	4	33	11–65	2	13	2–40	0.4
Muscle pain	2	17	3–49	2	13	2–40	1
Itch	1	11	0.4–40	5	31	12–59	0.2

CI: confidence interval.

^a^ Fisher’s exact test.

Among the patients with naïve infections, one female patient developed otitis media 1 week after infection and one child developed pneumonia 1 week after infection; both were treated with antibiotics.

### Comparison of laboratory parameters between subgroups

**Figure 4:**
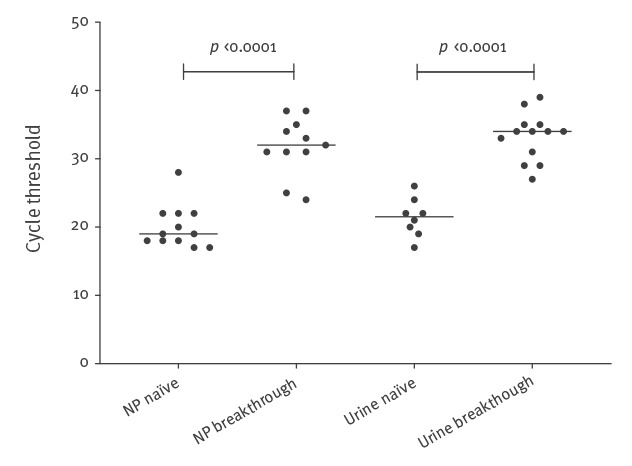
Cycle threshold values of measles real-time PCR in nasopharyngeal (n = 23) and urine samples (n = 21) in naïve and breakthrough measles infections in the Gothenburg area, Sweden, December 2017–January 2018

As shown in Table 1 and 2, measles virus RNA was detected in nasopharyngeal samples in all 12 patients with naïve infections with a median Ct value of 19 (IQR: 18–22). The Ct values in nasopharyngeal and urine samples were lower (indicating higher viral load) in subjects with naïve vs breakthrough infections (Figure 4). Five of 16 breakthrough infections were negative for measles RNA in nasopharyngeal samples. IgM and IgG levels at first sampling and at FU are presented in Table 1 and 2. IgG during the symptomatic phase was negative in all subjects with naïve infections except for Patient 12. Two cases with breakthrough infections had IgG levels below the cut-off value. Both cases reported previous vaccination against measles.

NP: nasopharyngeal sample.

Black line: median value. P values indicate significant differences between groups.

### Avidity of measles virus specific IgG antibodies

Avidity analysis of IgG antibodies was performed on all samples where sufficient material was available (16 acute and 19 follow-up serum samples). All 13 cases with breakthrough infections tested had high-avidity IgG antibodies in the acute serum sample. Only one patient with naïve infection had detectable IgG antibodies in the acute serum sample. These antibodies were of low avidity. Results of IgG avidity testing are presented in Tables 1 and 2.

### Outbreak control measures

All 125 suspected cases of measles underwent clinical evaluation and sampling at Sahlgrenska University Hospital. Altogether, ca 3,000 potentially exposed individuals were notified by letter or phone call. More than 1,000 doses of post-exposure measles vaccine and 300 doses of post-exposure immunoglobulin were administered according to Swedish recommendations [4].

Nine of the 28 confirmed cases were HCW exposed to measles through close contact with patients in their clinical work and all were breakthrough infections. They reported previous measles vaccination (five had two and one had at least one documented vaccine dose, whereas three reported at least one vaccine dose although not documented). We did not identify onward transmission from any of the HCW with measles.

### Transmission and secondary cases

We identified five cases (all with naïve infections) who caused onward transmission (Table 1 and 2). Two of these (Patients 1 and 3), both with a high viral load in nasopharyngeal samples (Ct values of 18 and 17, respectively), transmitted measles to 24 persons. Fourteen of these 24 transmissions occurred in hospital settings (Figure 2). We did not identify any onward transmission from breakthrough infections.

## Discussion

Our observation is in accordance with other recent reports of outbreaks in areas with high vaccination coverage [11,25]. We propose that individuals with breakthrough infection of measles can be identified with a fast provisional classification using routine laboratory testing of IgG at or after onset of rash. This was confirmed by IgG avidity testing of acute serum samples in our study. Earlier reports from outbreaks of measles have described breakthrough infections in individuals with previous vaccination against measles [5-10,14,26]. No onward transmission from breakthrough infections was identified in our study. This is in line with other reports which also indicate that breakthrough infections rarely cause onward transmission [13,14]. Cough was less common in breakthrough infections which, together with lower levels of viral RNA, may explain the limited infectiousness of breakthrough infections. As far as we know, there are very few published cases in which transmission from breakthrough infections is likely to have occurred, all after prolonged close contact among household members [13,15]. Rosen et al. reported transmission of measles from a twice-vaccinated individual but the details in their report suggest that their case probably was a primary vaccine failure [26].

All but two of our patients with breakthrough infections had IgG antibody levels at the onset of rash that were above the defined protective level, indicating pre-existing immunity. In addition, they all developed a rapid secondary IgG response. The two patients who did not have IgG at the onset of rash had at least one documented dose of measles vaccine and both had low measles RNA levels as well as a mild clinical course, supporting the conclusion that they indeed had breakthrough infections.

The reason why some patients with previous vaccination develop symptomatic infection is not known, but the lack of natural boosting in the community might be of importance [27]. The viral genotype (B3) and the level of exposure could also be contributing factors [28]. Nevertheless, the presence of pre-existing immunity probably contributed to a milder clinical course compared with patients with naïve infections in our study. This is in line with the recent report by Cherry et al., who found a milder clinical course in individuals with breakthrough measles, especially if they had received two or more doses of measles vaccine [13].

PCR has been used for the diagnosis of measles since more than a decade [23]. Our data indicate that patients with breakthrough infections seem to present with lower levels of measles RNA at rash onset, most probably because partial immunity reduces viral replication. By contrast, those with naïve infections seem to have a high viral load [18] and are more likely to transmit the infection. Even though the measurement of measles RNA load is semiquantitative, a Ct value of 18 in a nasopharyngeal sample, as in the index case in the outbreak described here, translates to a viral load that is ca 10,000 times greater than that of Ct 32, as in the cases with breakthrough infections. Nevertheless, Santibanez et al. recently reported, although not presenting Ct values, onward transmission from a breakthrough infection to a household member [15]. It is noteworthy that two patients in our study transmitted the infection to the majority of the other cases in the outbreak. Both had very high measles RNA levels in nasopharyngeal samples taken at onset of rash, which is in line with another recent report [18].

In five of the six cases with vaccine infections, measles RNA could be detected in nasopharyngeal samples. Interestingly, IgG antibodies were lacking in samples taken at the onset of rash, and the severity of symptoms was similar or more pronounced than in patients with breakthrough infections. It is therefore essential that, during an ongoing outbreak, information on recent MMR vaccination is recorded and that genotyping of the detected virus is performed if a vaccine infection is suspected [29].

It is not possible to distinguish breakthrough measles from naïve infections using clinical criteria. We therefore suggest that individuals with and without pre-existing immunity can and should be identified during an outbreak using laboratory criteria. Contact tracing around breakthrough infections can, most probably, be limited to individuals with prolonged close contact, such as household members and especially vulnerable individuals, for example patients with immunosuppression. We also suggest that healthcare authorities review published data on measles breakthrough infections and take these into account when revising public health guidelines.

This study was performed in an area with high vaccination coverage and the results may not be representative for populations with low immunisation rates where naïve infections are predominant. It has several limitations. The number of secondary cases may have been underestimated as some individuals with mild symptoms may have been missed during the outbreak. However, we have reason to believe that they were few, as the outbreak was widely reported in the media and a large number of individuals with mild symptoms were sampled and tested for measles. In several of the patients with breakthrough infection, the history of vaccination relied on self-reporting instead of written documentation, which means that there is some degree of uncertainty, but in these cases, IgG avidity testing and IgG levels in FU samples strongly support previous immunisation. Terms like modified measles, secondary vaccine failure and re-infection after remote vaccination have been used by other authors to describe breakthrough infections [9,10,22]. We propose a fast provisional classification, which might preclude comparison with previous reports. However, we believe that our classification, which is in line with the data presented by Hahne et al. [14], is concise and can guide initial decisions during outbreaks of measles.

Our proposed application of Ct values also has limitations. Ct values are only an estimate of viral load and can be influenced by poor sampling as well as differences between assays, such as real-time PCR efficiency and Ct value read-out settings. In our experience, technical issues usually change Ct values only by a few cycles, that is, much less than the difference in Ct value (10–15 cycles) that we observed between patients with high and low viral loads in our study. Still, the small number of cases makes it difficult to propose universal cut-offs for Ct values to distinguish naïve and breakthrough infections or levels that represent a significant risk of transmission.

Since all cases with breakthrough infection were sampled at or within 4 days after onset of rash we could not analyse viral loads in these individuals before onset of rash. Likewise, measles IgG levels before exposure in patients with breakthrough infection were not available.

## Conclusion

We show that there was a large difference in viral load in nasopharyngeal samples between patients with naïve and breakthrough infections of measles, and our results indicate that a high risk of onward transmission is confined to naïve infections. We propose a fast provisional classification of breakthrough measles that can guide contact tracing in outbreak settings.
